# The effects of threat on complex decision-making: evidence from a virtual environment

**DOI:** 10.1038/s41598-024-72812-2

**Published:** 2024-09-30

**Authors:** Aaron Laycock, Guy Schofield, Cade McCall

**Affiliations:** 1 Department of Psychology, University of York, York, YO10 5DD UK; 2 Department of Archaeology, University of York, York, YO10 5GB UK

**Keywords:** Threat, Virtual reality, Complex decision-making, Computational modelling, Iowa gambling task, Choice perseveration, Reward sensitivity, Computational models, Human behaviour

## Abstract

Individuals living and working in dangerous settings (e.g., first responders and military personnel) make complex decisions amidst serious threats. However, controlled studies on decision-making under threat are limited given obvious ethical concerns. Here, we embed a complex decision-making task within a threatening, immersive virtual environment. Based on the Iowa Gambling Task (IGT), a paradigm widely used to study complex decision-making, the task requires participants to make a series of choices to escape a collapsing building. In Study 1 we demonstrate that, as with the traditional IGT, participants learn to make advantageous decisions over time and that their behavioural data can be described by reinforcement-learning based computational models. In Study 2 we created threatening and neutral versions of the environment. In the threat condition, participants performed worse, taking longer to improve from baseline and scoring lower through the final trials. Computational modelling further revealed that participants in the threat condition were more responsive to short term rewards and less likely to perseverate on a given choice. These findings suggest that when threat is integral to decision-making, individuals make more erratic choices and focus on short term gains. They furthermore demonstrate the utility of virtual environments for making threat integral to cognitive tasks.

## Introduction

Some of life’s most difficult decisions are made within dangerous environments. Indeed, individuals such as first responders or military personnel make critical, complex decisions amidst threats to life and limb^1–4^. Nevertheless, many questions remain regarding the effects of threat on complex decision-making^5^. Research in this area furthermore presents considerable methodological challenges in terms of both manipulating threat and measuring decision-making’s underlying processes.

Complex decisions are characterized by uncertainty and the need to balance multiple competing goals^6^. This combination presents a significant cognitive challenge that is likely amplified in the presence of threats. In the most basic sense, complex decisions require attention, which itself is shaped by threat in many ways^7^. Furthermore, optimal decision-making frequently requires an individual to apply their existing knowledge and to learn about the given situation. These processes are also likely influenced by threat, given its effects on memory^8^ and learning^9^.

More specifically, complex decision-making often involves reinforcement learning; optimal choices depend upon accommodating for feedback from the environment^10^ and from the outcomes from one’s choices^11^. Threat may disrupt this ability to flexibly respond to changes in rewards and losses over time. For example, research demonstrates that under threat of shock, participants are slower to switch away from disadvantageous choices following negative feedback^12^. Other work suggests that threatened individuals are less likely to explore their options when problem-solving^13^ and instead employ simple heuristics^14^.

Despite these clues that threat negatively affects many of the cognitive processes underlying complex decision-making, research directly examining the effects of threat is limited and the findings are mixed^15^. This inconsistency may in part be due to fundamental differences in decision-making paradigms. Some work focuses on decisions from description, where participants respond to hypothetical questions (e.g., participants are asked, “If X happened, how would you respond?”), while other work focuses more directly on decisions from experience (e.g., participants are put in X situation and must make an actual decision). These different approaches sometimes yield different conclusions^16^. For example, research using decisions from description supports the idea that individuals overweigh low probability events when making risky decisions^17^, while research using decisions from experience suggests that individuals actually underweigh them in those circumstances^18^.

Research on decisions from experience has frequently used multi-armed bandit tasks^19^, tasks in which participants’ decisions incur costs and benefits. The Iowa Gambling task (IGT^20^), for example, requires individuals to select cards from four separate decks over a sequence of trials. Each selection leads to losses and gains, usually in financial terms. Initially, individuals have no information about the probability of payoff from the decks. Instead, they gain information from sampling the decks across trials. Maximising net reward depends on an individual’s ability to learn which decks are most profitable over time and to adapt their choices accordingly. Performance on the this task is ostensibly representative of real-world decision-making and its underlying cognitive processes^20,21^. When it comes to the effects of threat on multi-armed bandit tasks, findings are mixed. Some studies find that threat or acute stress decreases overall performance^22^, while others find it can improve aspects of decision-making^23^. Still others find no effect at all^24^.

This diversity in results is perhaps not surprising given the variety of methods used across studies^5^. Critically, threat is manipulated in different ways, often via a task that is incidental to the decision-making task itself. For example, some studies have examined the effect of threat on IGT performance by manipulating anticipatory stress; participants complete the decision-making task while knowing that afterwards they will be asked to complete a public speaking task^22,25–27^. Other studies have participants complete a decision-making task after a stressful experience, such as a cold pressor task^28^.

The manipulations in these examples are indeed effective, eliciting both subjective and physiological responses and oftentimes affecting decision-making performance^22,25–27^. Nevertheless, the relationship between the threat and the decision-making task in these paradigms is incidental; the outcome of decisions is not directly related to the outcome of the threat. While these incidental manipulations of threat can elicit a threat response, they may only tell us about situations where threat is a distractor (i.e., situations in which optimal performance might rely upon ignoring the threat). But they may not tell us about situations when threat is integral to the decision-making task (i.e., when optimal performance determines success in dealing with the threat). This distinction is likely critical in the “real world”. For example. threat might act as a distractor for a military medic making decisions about how to deliver care to a patient in the midst of a hostile environment. On the other hand, threat is integral to decision-making when that medic is choosing the safest route out of hostile environment. In this sense, real world threats might play a very different role depending on whether they are incidental or integral to the decision at hand.

Virtual reality provides one means of creating paradigms where threat is integral to decision-making. Virtual environments can elicit subjective and physiological responses via simulations of threats such as being perched precariously on the edge of a cliff or being surrounded by dangerous animals^29–31^. They can also immerse individuals in ambiguously threatening environments which create a prolonged experience of anxiety and unpredictability^32^. As such, they potentially allow researchers to create decision-making tasks in which one’s decisions are integrally connected to threats in the surrounding environment.

Another potential cause for the diversity of results in the threat and decision-making literature is the disparity in decision-making measures. Multi-armed bandits have been used to examine learning from feedback, loss aversion, risk taking, and other features of complex decision-making that are potentially affected by threat. Even within a given paradigm, approaches differ. With regards to the IGT, measures have evolved over time. Performance was initially quantified by simply calculating the ratio of advantageous over disadvantageous choices^20,33^**.** More recent research has shifted the focus to the cognitive processes underlying those choices via computational modelling of the behavioural data^34–36^. Depending on the nature of the model, these methods derive a range of parameters that reflect learning and decision-making mechanisms such as reward sensitivity, loss aversion, and choice perseveration.

Recent work along these lines suggests that threatened individuals may be less responsive to feedback from their choices in the IGT^37^. Although the detrimental effect of threat on overall IGT score reported in previous studies^22,27^ was not replicated in Ben Hassen and colleagues’ study^37^, their computational modelling suggests that threatened participants were less sensitive to feedback. However, the particular computational model used in their study did not distinguish between loss aversion and sensitivity for reward, which might be critical in our understanding of the general effects of threat^23,38^. The novel Outcome-Representation Learning model (ORL)^34^ addresses this concern. Developed to reflect the cognitive strategies that underpin performance on the IGT, the ORL has separate parameters for loss sensitivity, reward sensitivity, and reward frequency sensitivity. Moreover, the ORL also has parameters for memory and choice perseveration, key features of complex decision-making that the abovementioned research suggests could be influenced by threat.

In the studies presented here, we sought to address open questions regarding the effects of threat on complex decision-making. Specifically, we sought to test if a threat that is integral to the decision-making process would lead to decrements in decision-making performance. We also sought to test if, as the literature suggests, threat would lead to a change in reward or loss sensitivity^37^ as well as a reduced tendency to explore the range of options^12–14^. To do so, we developed a VR-based complex decision-making task structured like the IGT, in which decisions were tied directly to threats in the environment. We furthermore used the ORL computational model to explore the effects of integral threat on cognitive underpinnings of complex decision-making.

## Study 1

The aim of Study 1 was to pilot a virtual world for observing complex decision-making in a naturalistic and potentially threatening environment. We embedded a task based on the IGT within a virtual reality environment (the VRIGT). We then tested whether performance would improve over the course of trials, in line with the traditional IGT^20^. We also tested whether the ORL computational model used to fit IGT data would also work with VRIGT data.

### Method

#### Participants

Seventy-one (71) participants were recruited to take part in Study 1. One participant was excluded due to medical concerns. Of the full sample (70), 58 participants (34 females, 24 males) completed all measures without technical difficulties. All participants were over 18 years old. Participants were given the opportunity to receive either course credit or a six-pound gift card as reimbursement for their time. Approval for the data collection was granted by the ethics committee of the Psychology Department at the University of York, and all experiments were performed in accordance with relevant guidelines and regulations.

#### VRIGT

We developed the VRIGT on the gaming development platform Unity (version 2020.3.15f2) using the SteamVR plugin (version 2.7.3). Some materials were imported from the Underwood Project^32^. The VRIGT was constructed around three scenes: a pre-scene (for practice and instructions), a test-scene (task) and an end-scene (end task).

The VRIGT test-scene was based on the Iowa Gambling Task^20^. In each trial of the classic IGT, participants select a card from one of 4 decks (A, B, C or D). Each turn of a card results in losses and or rewards. While the amount returned from any given deck changes between trials, some decks offer greater rewards over time. Success on this task relies on the ability to identify the pattern of reward and adapt choice accordingly^39^.

**Fig. 1:**
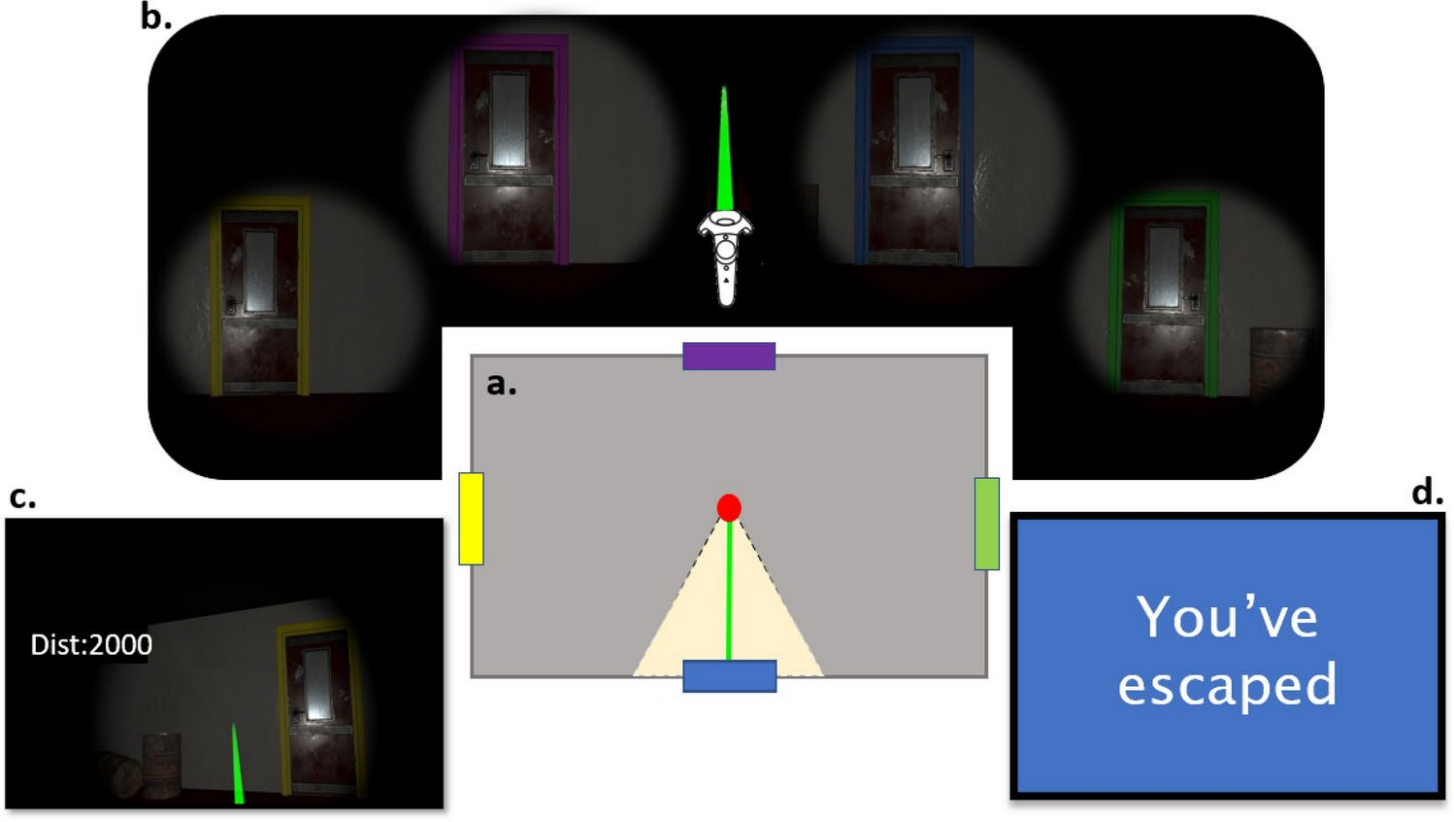
Illustrations of the VRIGT Test Scene. (**a**) During the VRIGT test-scene, participants are orientated in the centre of a single room and have 360° freedom to rotate. Colour-coded doors are presented at the centre of each of the surrounding walls. The red dot represents the players’ location. (**b**) Door choices are made by placing the raycaster (green laser graphic) over the appropriate door and selecting using the trigger button. (**c**) At all stages during the test-scene, participants can view their current distance from the danger zone (index of reward) on a visible graphic. (**d**) At the end of the test-scene (i.e., after 80 trials), participants are instructed that they have safely escaped the building (end-scene).

In Study 1’s version of the VRIGT, participants are told that they are in a building that is slowly collapsing, and their goal is to maximise distance from a danger zone. On each trial they enter a new room. Their task is to choose which door they will use to exit that room. These doors (4 doors = A, B, C and D) replace the decks of the IGT; the financial rewards of the IGT are replaced with meters from the danger zone. In other words, each door selection changes the participant’s distance from the danger zone. Participants see their current distance via a display located within their view (see Fig. 1c).

Scoring starts at a distance of 2000 m from the danger zone (as opposed to $2000 facsimile US bills in the traditional IGT)^20^. The specifics of each selection, e.g., “You gained 100 m from the danger zone, but you lost 250 m”, are also played via an audible recording. This recording is paired with a 4-s visual fade to darkness, which alerts the participants that a selection has been made. As with the classic IGT, the VRIGT measures the participant’s ability to identify the pattern of rewards and losses (e.g., the door that statistically offers the largest distance increase from the danger zone) and to adapt choice accordingly. In study 1, the VRIGT test-scene consisted of 80 trials (as in^40^). We chose the 80 trial version of the IGT instead of the original 100 trial version to avoid any fatigue that might arise from wearing the HMD. These 80 trials were subdivided into four 20-trial blocks for analysis.

Doors in the VRIGT were colour-coded to ensure that participants recognized them from trial to trial (see Fig. 1b). Two versions of the VRIGT were created to counterbalance door colour. As with IGT decks A and B, VRIGT doors A and B are low-scoring in the long run. Although selecting these doors offers the highest maximum per-trial reward (100 m gained from the danger zone), choosing these doors leads to greater losses over time. Doors A and B are identical in sum of losses but differ in frequency of loss. Door A has a series of regular small losses, whereas Door B is associated with rare but large losses. As with decks C and D in the IGT, doors C and D return a low reward initially (50 m gained), yet the loss over time is less. The frequency of loss received from selecting doors C and D are matched to doors A and B, respectively. The scoring matrix used in the VRIGT is based on the original IGT (as used in^41^).

Study 1’s version of the VRIGT was made to be ambiguously threatening following the approach of McCall et al.^32^. Following every door selection, the volume of a background audio clip (of a building demolition) increased by a small amount. Lighting was dim, the air appeared dusty (via a particle system), and participants used a torch to illuminate the room.

#### Hardware

Participants experienced the immersive virtual environment via a VIVE head-mounted display (HMD) with an integrated Dual AMOLED 3.6’’ diagonal screen, a resolution of 1080 × 1200 pixels per eye (2160 × 1200 pixels combined), refresh rate of 90 Hz, and a 110 degrees field of view. Participants also used a wireless VIVE controller with dual-stage trigger and integrated HD haptic feedback. For audio, participants wore wireless headphones set to a maximum volume of 80%.

#### Questionnaires

In the pre-task questionnaire, participants were asked about their age, gender, and video game experience. For exploratory purposes, participants also completed individual difference scales related to intolerance of uncertainty; these are not reported here. In the post-task questionnaire, participants completed a series of questionnaires about their experience in the VRIGT. To rate subjective experience of affect, participants used a slider (ranging from “not at all = 0” to “a great deal = 100”) to rate the extent to which the VRIGT was: “frightening”, “creepy”, “unpredictable”, “amusing”, “funny”, “engaging”, “confusing”, “disgusting”, “interesting”, “surprising”, “frustrating”, “sad”, “boring”, and “enjoyable”^32^. Participants also completed user engagement^42^, and tension^43^ scales (see Supplementary Materials for details).

#### Procedure

After providing informed consent and completing the pre-task questionnaire. Participants were then told what to expect over the course of the task. This included information regarding the VRIGT layout and how to make selections with the controller. Participants were then helped into the HMD and were introduced to the virtual world. We counterbalanced the starting orientation between conditions across four possible orientations. This was done to control for any bias resulting from the spatial start location**.**

The initial VRIGT pre-scene provided participants with instructions and an opportunity to practice using the controller. Participants were instructed to identify all four doors in the visual display, confirm that the audio level was sufficient, and make a door selection using the appropriate trigger located on the controller. At the end of the pre-scene, participants listened to a set of instructions presented within the virtual world that provided the task narrative, aims, and rules (see Supplementary Materials). Participants then completed the task itself for 80 trials (door selections).

After the trials were completed the task ended and participants were told that they had successfully escaped the building. Participants then completed the post-task questionnaire. Participants were then debriefed and given information about payment or course credit.

### Analysis

#### Performance during the VRIGT

As with the traditional IGT^20^, performance was calculated as a difference between advantageous and disadvantageous selections (C + D) − (A + B). Positive scores (> 0) demonstrate an overall trend of selecting doors that minimise net loss. We calculated an overall score (all 80 trials) as well as a score for each 20-trial block, as is the convention^44^. In the traditional IGT, participant performance improves over the blocks^45^ with improvement expected after about 40 trials in non-clinical populations ^46^. This increase reflects participants learning to discriminate between advantageous and disadvantageous choices over consecutive trials^20^.

#### Computational modelling

We also tested whether the ORL computational model used to successfully model IGT data in prior research would also fit our VRIGT data. Prior research^34^ tested the ORL’s model performance (e.g., short- and long-term prediction accuracy and parameter recovery) using data from multiple IGT studies with diverse samples. The ORL showed comparable or better performance than other models used to analyse IGT data.

Moreover, the ORL provides parameters that are theoretically important for understanding the influence of threat on complex decision-making. (1) Reward sensitivity (Arew), where higher values represent a greater influence of reward on learning (from 0 to 1); (2) loss sensitivity (Apun), where higher values represent a greater influence of punishment on learning (from 0 to 1); forgetfulness (K), which represents how quickly decision makers forget their past choices (from 0 to 242), with greater values representing shorter retention; reward frequency sensitivity (betaF), in which selections are based on win frequency (from—∞ to + ∞) and positive values demonstrate a preference for options with a high win frequency; 5) choice perseveration (betaP), where higher values reflect a tendency to repeatedly select from the same option (from—∞ to + ∞).

To confirm that the ORL had at least equivalent fit for VRIGT data as other IGT-related computational models, we also tested the Prospect-Learning Valence Delta model^47^, the Prospect-Learning Valence Decay model^48^, the Value-Plus-Perseverance model^49^, and the Outcome Representation Learning model^34^.

Modelling was run using the “hBayesDM” in R^50^, following Haines et al.^34^. All models were sampled for 4000 iterations, with the first 1500 as warmup (i.e., burn-in) across four sampling chains (10,000 posterior samples for each parameter total). Model convergence was judged visually by inspection of trace-plots and assessment using the Gelman–Rubin test^51^, $\hat{R}$ values < 1.1 suggest adequate model convergence.

#### Software

All analyses were done in RStudio 4.2.1 [64]^52^, using R basic or the “lme4” 1.1-26 package^53^. For all LMMs, p values were calculated using the “lmerTest” package 3.1-3^54^ and the ANOVA function using Satterthwaite’s method for F tests. Pairwise post hoc comparisons were calculated using the “emmeans” package 1.6.3^55^. All pairwise post hoc comparisons used a Tukey correction for p-values. A MVT correction was applied when adjusting for multivariate comparisons. The “bayesplot” package was used visualise posterior predictive checks^56^. Finally, univariate outliers were identified using the median absolute deviation (MAD) using the “routliers” package^57^.

### Results

Regarding previous gaming experience, the sample was generally balanced: none = 19 (26.76%), some = 31 (45.07%), and lots = 20 (28.16%). See Supplementary Materials for details of reported user engagement, tension, and subject experience (see Supplementary Fig. 3) during the VRIGT.

#### Performance during the VRIGT

**Fig. 2:**
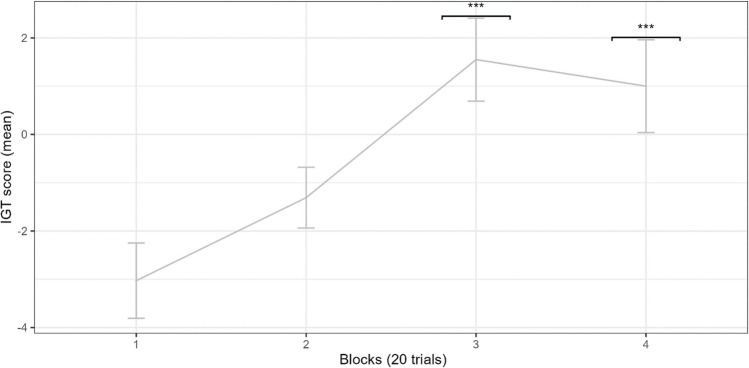
Performance over time. Error bars represent −/+ standard error. *Indicates differences from baseline with a significant p value * < 0.050, ** < 0.01, *** < .001.

Performance scores (M = − 1.79, SD = 16.72, 95% CIs: − 6.19, 2.60) were normally distributed. Using the threshold of 3* MAD (+/− median), no outliers were identified. To model the effect of performance over time, we used a linear mixed-effects model (LMM) using block as a fixed factor and performance score as the dependent variable. Block 1 was used as the reference level. Intercepts were allowed to vary as a random factor at the level of the individual. Results demonstrate (see Supplementary Table 2) that the effect of block on performance was significant (F(3, 171) = 9.26, *p* > 0.001). Performance improved as blocks progressed. Performance in blocks 3 (t.ratio (171) = 4.65, *p* < 0.001) and 4 (t.ratio (171) = 4.09, *p* < 0.001) was significantly better than block 1 (see Fig. 2). Post hoc comparisons between sequential blocks show that the performance significantly improved between blocks 2 and 3 (t.ratio (171) = − 2.90, *p* = 0.022, all other *p*’s > 0.05). As with the traditional IGT^44,46^, these results demonstrate that at group level, the ability to discriminate between advantageous and disadvantageous choices improved after approximately the 40th trial.

#### Computational modelling

As with prior research on the IGT, each of the computational models we tested had adequate model convergence with all $\hat{R}$ values < 1.1. The best model fit (see Supplementary Table 1), however, was with the Outcome Representation Learning model^34^ (see Supplementary Figs. 1 and 2 for posterior distributions of the hyper (group) parameters and posterior predictive checks).

## Study 2

Building on the findings of Study 1, we developed threatening and nonthreatening versions of the VRIGT. This allowed us to test if integral threat affects learning and overall performance in a complex decision-making task. We also used the ORL computational model to test for any effects of threat on key parameters in the decision-making process (e.g., reward sensitivity, loss sensitivity, choice perseveration, and etc.). The research questions and analyses for this study were preregistered [As predicted: 130526].

### Methods

#### Participants

One-hundred (100) participants (male = 46, female = 51, non-binary = 3) with an average age of 20 (M = 20.84, SD = 4.23) participated in Study 2. This sample size was chosen based on power analyses using the “smir” package in R^58^; see Supplementary Materials for details. Participants reported a range of gaming experience, none = 13%, some = 53%, and a great deal = 34%. Participants were randomly allocated into either the threatening (n = 50) or nonthreatening (n = 50) experimental conditions. Approval for data collection was granted by the ethics committee of the Psychology Department at the University of York and all experiments were performed in accordance with relevant guidelines and regulations.

#### Materials

The materials used in Study 2 were similar to those of Study 1 with a few changes. Study 2 used two versions of the VRIGT, a threatening version and a nonthreatening version. In the threatening version, participants were told that their task was to escape a collapsing building. In the nonthreatening version, participants were simply told that their task was to exit an office building; there was no mention of any threats (transcripts available in the Supplementary Materials). We used these two versions in a between-subjects design which we chose to avoid practice effects from repeating the IGT^59^.

**Fig. 3:**
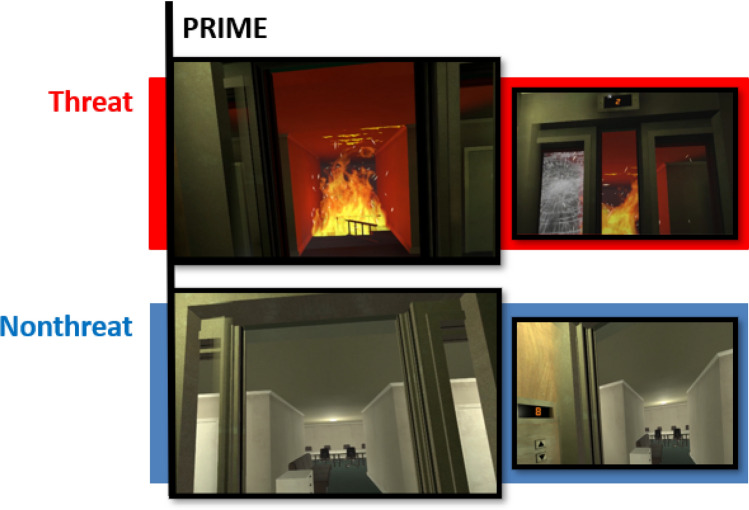
Screenshots of Study 2 prime-scenes for the two conditions.

In terms of the virtual world’s content, both VRIGT versions used in Study 2 included a prime-scene before the task (150 s). This prime-scene differed between conditions (see Fig. 3). In the threat condition, participants entered an elevator that gradually descended 8 floors. The elevator had transparent doors and on each floor the doors opened. Over the course of the descent, participants encountered threats that gradually increased in intensity. First, an audible warning instructed participants not to enter the lift (although they had no control in doing so). This was followed by an exploding light fitting, fire in the hallway opposite the elevator, cracking lift windows, further encroaching fire, and finally, smoke which filled the lift compartment (video in Supplementary Materials). The nonthreatening condition included a different prime-scene (also 150 s); participants still rode the elevator for 8 floors, but there were no threatening stimuli, they simply travelled past floors of a mundane office building.

We also changed the content of the task rooms slightly in Study 2. To give a sense of moving through a series of different rooms (as stated in the narrative), the appearance of the task room changed between trials for both conditions. These changes included subtle adaptations to the walls, floor, and lighting. In between trials, the time of the fade was extended to 4.8 s. There were also minor differences between conditions in the task scenes. Capitalizing on the threatening effects of darkness^60^, lighting in the threat condition was the same as Study 1 (i.e., dim with a torch), while rooms were more brightly lit in the nonthreatening condition. The threat condition also included ambient red lighting to signal danger^61^ and some aversive sound effects. These effects were taken from sounds already experienced by the participants during the prime-scene but were not paired with any visual effects. Because participants did not report fatigue in the 80 trial version from Study 1, we increased the number of trials during the task to 100, meaning that the test-scene did not end until these trials had been completed.

To measure differences in physiological arousal between conditions, skin conductance level (SCL) and heart rate (via ECG) were recorded using AcqKnowledge 5.0 software (Biopac Systems Inc., Santa Barbara, CA) and the Biopac MP160 acquisition system. SCL was recorded using a wireless Biopac BioNomadix amplifier (BN-PPGED). The electrodes were attached to the middle phalanges of the left middle and index fingers. Heart rate was recorded using a wireless Biopac BioNomadix ECG (BN-RSPEC) amplifier with a three-lead set. Electrodes were placed on the sternal end of the right clavicle, left mid-clavicle, and lower left rib cage. These physiological data were collected during a 5-min baseline before the task, during the prime scene, and during the task itself. Heart rate data were averaged over these blocks. For skin conductance data, we took the average skin conductance minus baseline over the given block. Due to a technical fault during recording, we excluded two participants’ SCL data from this analysis.

To test for differences in timing between conditions, we also recorded trial time length.

### Results

#### Manipulation checks

To test differences in subjective experience between the two conditions, we tested for group differences in the post-task questionnaire (see Supplementary Figs. 4 and 5). For the affect variables, we used a LMM to predict rating, with fixed factors for affective category (e.g., “unpredictable”), condition, and their interaction. Intercepts were allowed to vary as a random factor at the level of the individual. A significant effect of condition (F(1, 98) = 23.31, *p* > 0.001), and its interaction with affective category (F(13, 1274) = 7.44, *p* > 0.001) was found on rating (see Supplementary Table 3). Post hoc comparisons of affective terms suggested that between conditions, only ratings that the VRIGT was “frightening” (t.ratio (1179) =− 7.46, *p* < 0.001), “creepy” (t.ratio (1179) =− 6.51, *p* > 0.001), and “surprising” (t.ratio (1179) =− 3.42, *p* = 0.009, all other *p*’s > 0.05) were significantly different, with those in the threat condition reporting higher ratings in all instances (see Supplementary Table 4). The MVT correction was applied to adjust for multivariate comparisons.

We compared mean heart rate between conditions for the baseline, prime-scene, and task-scene. At baseline there was no significant difference in heart rate between the threatening (M = 89.77, SD = 15.62) and nonthreatening (M = 86.94, SD = 12.20) conditions, t (98) = − 1.01, *p* = 0.314. During the prime-scene, average heart rate was significantly higher in the threatening (M = 98.85, SD = 17.08) versus nonthreatening (M = 92.76, SD = 13.28) conditions, t (98) = − 1.99, *p* = 0.049. During the task-scenes, heart rate was also higher in the threatening (M = 101.29, SD = 17.04) versus the nonthreatening (M = 94.93, SD = 11.95) conditions, t (98) = − 2.16, *p* = 0.033.

We found no effect of condition on average SCL at baseline. There was also no difference between conditions in the baseline corrected averages for the prime or task scenes (all *p*’s > 0.050).

The average trial time taken (in seconds) on each trial during the task-scene was not significantly different between the threatening (M = 5.85, SD = 2.02) and nonthreatening (M = 5.47, SD = 1.33) conditions, t (98) = − 1.10, *p* = 0.273.

#### Performance

Performance scores were normally distributed, and no outliers were identified. A t test (two-tailed) comparing performance between threatening (M = − 13.04, SD = 29.13) and nonthreatening (M = − 0.92, SD = 22.22) conditions showed a significant difference, t (98) = − 2.15, *p* = 0.034. Participants performed worse in the threat condition.

**Fig. 4:**
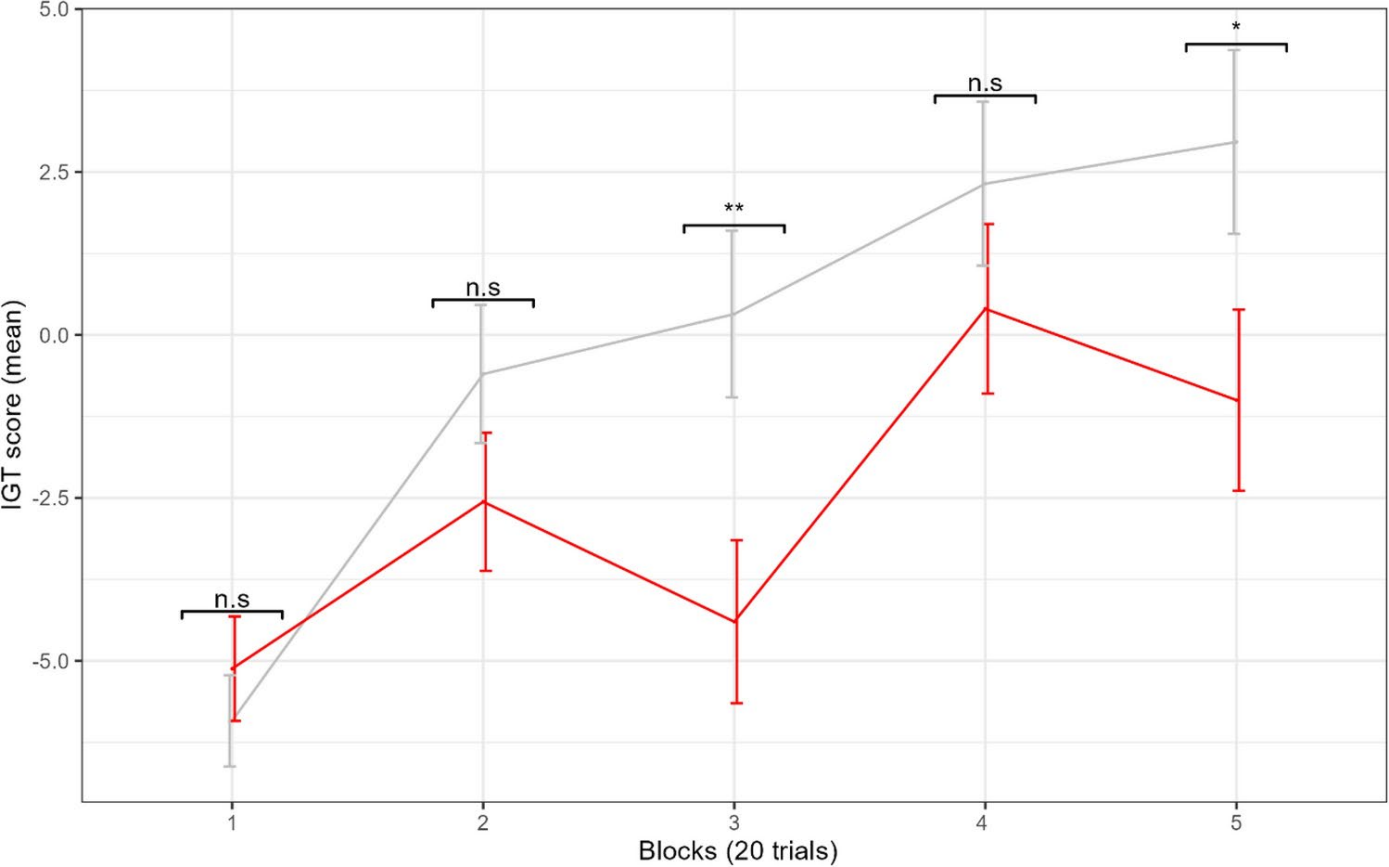
Performance over time between conditions. Conditions (red = threat, grey = nonthreat). Error bars represent −/+ standard error. *Indicates differences between conditions with a significant *p* value * < 0.050, ** < 0.01, *** < 0.001.

To assess performance over time, we ran an LMM with block, condition, and their interaction as fixed factors. Block 1 was used as the reference level. Intercepts were allowed to vary as a random factor at the level of the individual. Results demonstrate (see Supplementary Table 5) that the effect of condition (F(1, 98) = 4.62, *p* = 0.034), and its interaction with block (F(4, 392) = 2.47, *p* = 0.045) on performance was significant. A significant block by condition interaction on performance (see Fig. 4) was seen at block 3 (t.ratio (392) =− 2.87, *p* = 0.004) and block 5 (t.ratio (392) =− 2.47, *p* = 0.014, all other *p*’s > 0.05). This suggests that the differences in performance between the threatening and nonthreatening conditions first emerged at the point when non-clinical individuals in traditional IGT studies begin to migrate toward the more advantageous decks (i.e., around the 50th trial^46^) and the point at which meaningful individual differences tend to emerge^44^. While participants in the threat condition seem to close the gap by block 4, their learning is apparently limited. As a consequence, participants in the nonthreatening condition have a higher score in the final block when the benefits of learning peak^46^.

Breaking this down for participants in the nonthreatening condition only, we ran an LMM to assess the effect of block as a fixed factor on performance, with intercepts being allowed to vary as a random factor at the level of the individual. Results demonstrated a significant effect of block on performance (F(4,196) = 12.87, *p* > 0.001). Blocks two (t.ratio (196) = 3.84, *p* < 0.001), three (t.ratio (196) = 4.50, *p* < 0.001), four (t.ratio (196) = 5.94, *p* < 0.001), and five (t.ratio (196) = 6.40, *p* < 0.001) were significantly higher than block one (see Supplementary Fig. 6 and Table 6). Post hoc comparisons between successive blocks confirmed that performance significantly improved between blocks 1 and 2 (t.ratio (196) =− 3.84, *p* = 0.002, all other *p*’s > 0.05).

A different pattern of results emerged in the threatening condition. Here again we used an LMM to assess the effect of block as a fixed factor on performance, with intercepts being allowed to vary as a random factor at the level of the individual. Results demonstrated a significant effect of block on performance (F(4, 196) = 5.37, *p* > 0.001). Yet, only blocks four (t.ratio (196) = 3.87, *p* < 0.001) and five (t.ratio (196) = 3.09, *p* = 0.002) were significantly higher than block one (see Supplementary Fig. 7 and Table 7). Post hoc comparisons between blocks confirmed that performance only significantly improved between blocks 3 and 4 (t.ratio (196) =− 3.33, *p* = 0.009, all other *p*’s > 0.05).

#### Computational model

We applied the ORL, to the data from Study 2 for a more fine-grained analysis of behaviour across conditions (as in^34^). All $\hat{R}$ values < 1.1 suggest adequate model convergence (see Supplementary Figs. 9 and 11 for posterior predictive checks). Here, the posterior distributions from components of the ORL model were compared between the threat and nonthreat conditions of the VRIGT. This approach allowed for comparison between groups on model components, given that comparisons that do not include 0 (no change) within the 95% highest density interval (HDI) can be considered strong evidence in support of a difference^62^.

This approach revealed differences in reward sensitivity and choice perseveration between participants in the threat and nonthreat conditions of the VRIGT (see Table1). Participants in the threat condition (on average) had a greater tendency to update expectations after experiencing reward. They also switched between options (doors) more frequently than participants in the nonthreat condition. We found no robust support for differences between conditions in terms of loss sensitivity, frequency sensitivity, or forgetfulness.

**Table 1:** ORL group level comparisons. Means and SDs (in brackets) of the ORL model components.

	Threat	Nonthreat	95% HDI of comparison
Reward sensitivity	**0.28 (0.05)**	**0.08 (0.01)**	**0.107, 0.299**
Loss sensitivity	0.05 (0.01)	0.03 (0.01)	− 0.018, 0.041
Forgetfulness	0.87 (0.34)	1.51 (0.26)	− 1.487, 0.175
Frequency sensitivity	1.24 (0.21)	1.22 (0.18)	− 0.540, 0.578
Choice perseveration	**0.27 (0.66)**	**1.97 (0.57)**	− **3.481**, **− 0.046**

95% HDI based on the posterior distributions for the mean differences between groups. Bold indicates strong evidence of a difference between conditions.

## Discussion

Here we used a virtual reality-based version of the Iowa Gambling Task (IGT) to test the effects of threat on complex decision-making. We made threat integral to decision outcomes and tested its effects using both traditional measures of performance and computational modelling. In doing so, we show that threat reduces decision-making performance, likely by increasing focus on short-term rewards and decreasing meaningful choice perseveration.

In Study 1, we piloted our virtual reality version of the IGT (VRIGT). As with the traditional IGT, performance improved over the course of the task as participants learned to make more optimal choices. We were furthermore able to fit the data using computational models that have been useful for modelling IGT data in prior research^34^. Together, these findings are in line with previous work which shows that playing the IGT in a VR environment, when compared to a computerised desktop display, does not disrupt performance^41^. Moreover, participants’ subjective reports from Study 1 also suggest that the VRIGT provided a challenging and complex task.

In Study 2, we tested the effects of threat on complex decision-making performance and learning. We created two versions of the VRIGT, one threatening and one nonthreatening. The biggest differences between these conditions emerged before the task itself (i.e., in the prime-task scene), during which participants in the threat condition were immersed in a virtual building that was slowly collapsing. Participants in the nonthreatening condition were in a similar building but without the threatening details. This manipulation appears to have been effective, as participants rated the threatening environment as more frightening than the nonthreatening environment. Participants in the threatening condition also exhibited higher average heart rates throughout the experience (although we found no difference in skin conductance).

More importantly, the manipulation of threat negatively affected task performance overall and over time, with participants requiring more trials to significantly improve performance from baseline and scoring lower in the final block when compared with those in the nonthreatening condition. In general, these findings replicate previous work that demonstrated a negative impact of incidental threat on IGT performance and learning^22,26,27^**.** Our data add to this literature by showing these detrimental effects when threat is integral (and not incidental) to the decision-making task. Whereas prior work created situations where threat emerged as part of a separate task, divorced from the decision-making process itself, decisions in the VRIGT have direct relevance to threat imminence (i.e., on how far the participant is from danger).

To more closely investigate differences between conditions, we used the ORL computational model. These findings reveal that individuals in the threat condition were more driven by reward and displayed less choice perseveration (i.e., a greater tendency to switch between choices) over the course of the task. Indeed, these tendencies may be at the root of the poorer performance in the threat condition. Prior research has connected greater reward sensitivity with poor performance on the IGT^63,64^. The aim during the IGT (as is often the case in real-world situations) is to maximise net reward over time, which sometimes requires individuals to sacrifice immediate gain (e.g., the magnitude of a single trial) in favour of longer-term goals. Moreover, these findings support suggestions by Wemm and Wulfert^22^ that acute stress enhances the salience of reward-associated behaviours. Here, a bias to only focus on the metaphorical “carrot” and neglect the “stick” leads to sub-optimal performance.

These data are also roughly in line with Ben Hassen et al.^37^, who suggest that threat reduces loss aversion in the IGT, as evidenced by a lower index of the loss aversion parameter of the VPP computational model following threat manipulations. However, as noted by the authors, the VPP models loss aversion and sensitivity for reward as a single parameter (e.g., losses relative to gains). Therefore, the reported changes in this parameter in their study could be interpreted either as higher sensitivity to gains or as lower sensitivity to losses. We provide a degree of clarity here. We used the ORL model, which separates sensitivity for reward and loss^34^, and found that threat did not disrupt loss sensitivity, but did affect reward sensitivity.

The way individuals sample information via choice switching also tells us something about how they deal with complex decisions^10^. Some previous research suggests that when making decisions from experience, individuals in threat-related states sample more information before making a choice^65^. Yet these findings are at clear odds with suggestions elsewhere in the literature that incidental threat promotes premature closure, whereby an individual perseverates on a given choice before sufficiently exploring their options^13,66^. Here, using a task in which threat is integral to decision-making, we find that threat increased individuals’ tendency to switch between choices. That is, rather than early choice perseveration and limited exploration, threatened participants continued sampling from the different options. However, this greater exploration did not lead to improved performance. Instead, participants in the threatening condition performed worse than controls. With this in mind, the threatened group’s switching between options may have been more impulsive than strategic^37^. Future research could more directly test this claim with paradigms that evaluate impulsivity (e.g.,^67^). Moreover, future research could also directly test the effects of integral versus incidental threat on complex decision-making. Factors such as choice perseveration may be different when threat is an incidental distractor versus when an individual’s decisions have direct implications for the level of threat.

The findings presented here demonstrate the utility of combining existing decision-making paradigms with VR’s ability to effectively make threat an integral part of a task. Here, we used the IGT as our starting point. Future work could take a similar approach with other multi-armed bandit paradigms. Further work could also look at variability in the effects of threat on performance based on individual differences. Indeed, decision-making is shaped by many factors including other forms of affect^68,69^, working memory^70^, and intolerance for uncertainty^71^.

Regardless, the current findings suggest that when threat is an integral part of complex decision-making, it can disrupt learning from feedback, focus attention on short term rewards, and reduce perseveration on adaptive choices. This pattern of effects may have real world implications for individuals living and working in threatening environments. Threat biases the decision-making process; knowing the nature of those biases might help people in dangerous settings keep themselves and others safe.

## Supplementary Information

Supplementary Information.

## Data Availability

Data, analysis code, and supplementary materials are available on the OSF repository, https://osf.io/jg2qv/?view_only=0d42f9fce5d0466685e205fde92354d2.
