# Supplementary material for: The effects of threat on complex decision-making: evidence from a virtual environment
Source: Sci Rep. 2024 Sep 30;14:22637. doi: 10.1038/s41598-024-72812-2 (PMC11442743; doi:10.1038/s41598-024-72812-2)
Supplement: Supplementary file 1 — Supplementary Information. [file 41598_2024_72812_MOESM1_ESM.pdf]

The effects of threat on complex decision-making: Evidence from a virtual environment
Supplementary Materials

Contents

Study 1 3
Computational modelling 3
Model fits 3
Figure: Posterior distributions of the hyper (group) parameters 4
Figure: Posterior predictive checks 5
Subjective Experience 6
Affect 6
User Experience Scale 7
Tension 7
Performance 8
LMM Results: Performance over time. 8
Study 2 9
Videos 9
Power analysis 9
Pre-registration 9
Pre-scene instructional scripts 9
Threat script 9
Nonthreat script 10
Subjective Experience 11
Affect: between condition 11
Affect: within condition 13
User Experience Scale 15
Tension 15
Performance 16
LMM Results: Performance over time by condition 16
Figure: Nonthreat condition, Performance score over time. 17
LMM Results: Performance over time in nonthreatening condition 18
Figure: Threat condition, Performance score over time. 19

LMM Results: Performance over time in threatening condition 20
Computational modelling 21
Figure: Nonthreat condition, posterior distributions of the hyper (group) parameters .. 21
Figure: Nonthreat condition condition, posterior predictive checks 22
Figure: Threat condition, posterior distributions of the hyper (group) parameters 23
Figure: Threat condition, posterior predictive checks 24
Miscellaneous 25
OSF link 25
References 26

Study 1

Computational modelling

Model fits

Model	LOOIC
Prospect-Learning Valence Delta	12230.95
Prospect-Learning Valence Decay	11808.59
Value-Plus-Perseverance	11214.69
Outcome-representation learning	11173.73

Supplementary Table 1. LOOIC, Leave-one-out information criterion. ORL was the best fitting model in Study 1 using the LOOIC.

Figure: Posterior distributions of the hyper (group) parameters

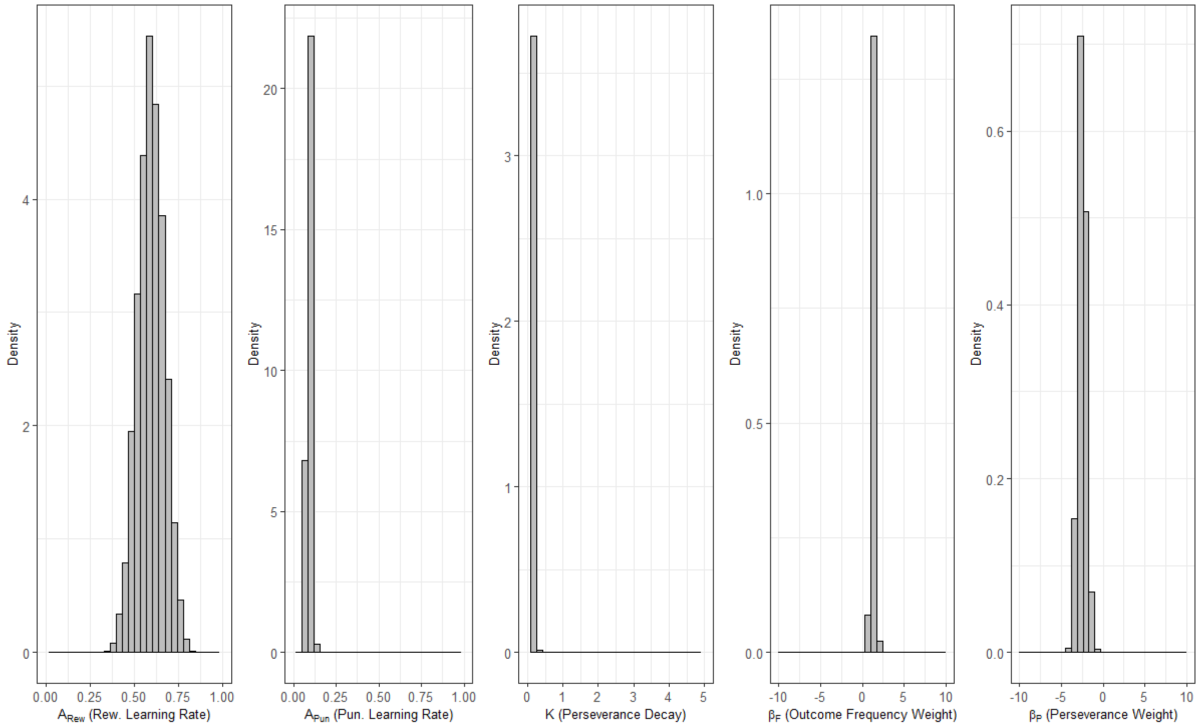

Supplementary Figure 1. Arew (Reward sensitivity), Apun (Loss sensitivity), K (Forgetfulness), betaF (Frequency sensitivity), betaP (Choice perseveration).

Figure: Posterior predictive checks

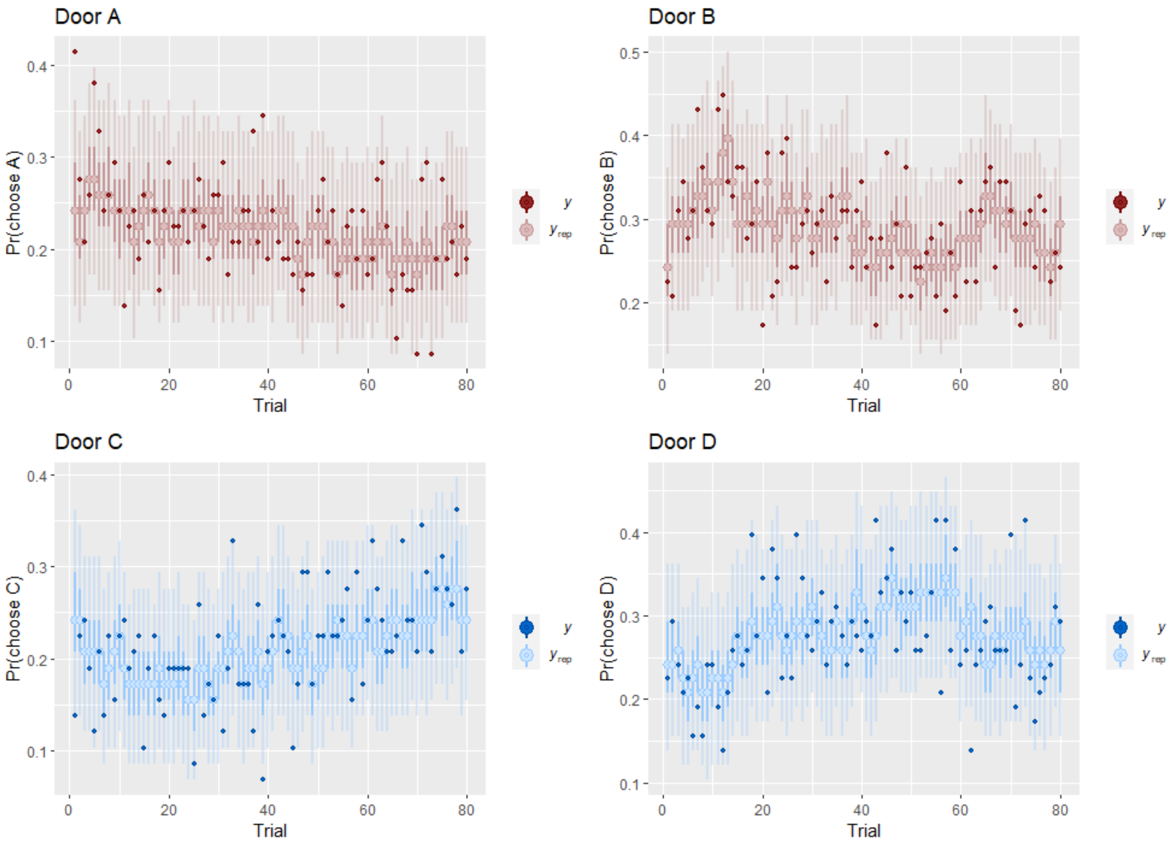

Supplementary Figure 2. Observed versus the posterior predicted probability of choosing each deck over trials. To illustrate performance trends “disadvantageous” doors are presented as red and “advantageous” doors in blue. The x-axis represents trial number, and y-axis the proportion of participants who selected the deck on each trial (Y), along with predictions (Yrep) computed from draws from the posterior predictive distribution of the same values. 50% (dark) and 95% (light) prediction intervals illustrate uncertainty.

Subjective Experience

Affect

We used a LMM to analyse the relationship between the affect terms participants used to report their overall feeling during the VRIGT. The model predicted rating with a fixed effect of affect category (e.g., "frightening"). Intercepts were allowed to vary as a random factor at the level of the individual. The affect category with the lowest average rating score ("disgusting") was used as a reference level. A significant effect of affect category was found ($F(13, 897) = 45.21$, $p < .001$). Pairwise comparisons are reported using the Tukey correction for p-values highlighted significant contrasts between affect categories. Results displayed in Figure 1.

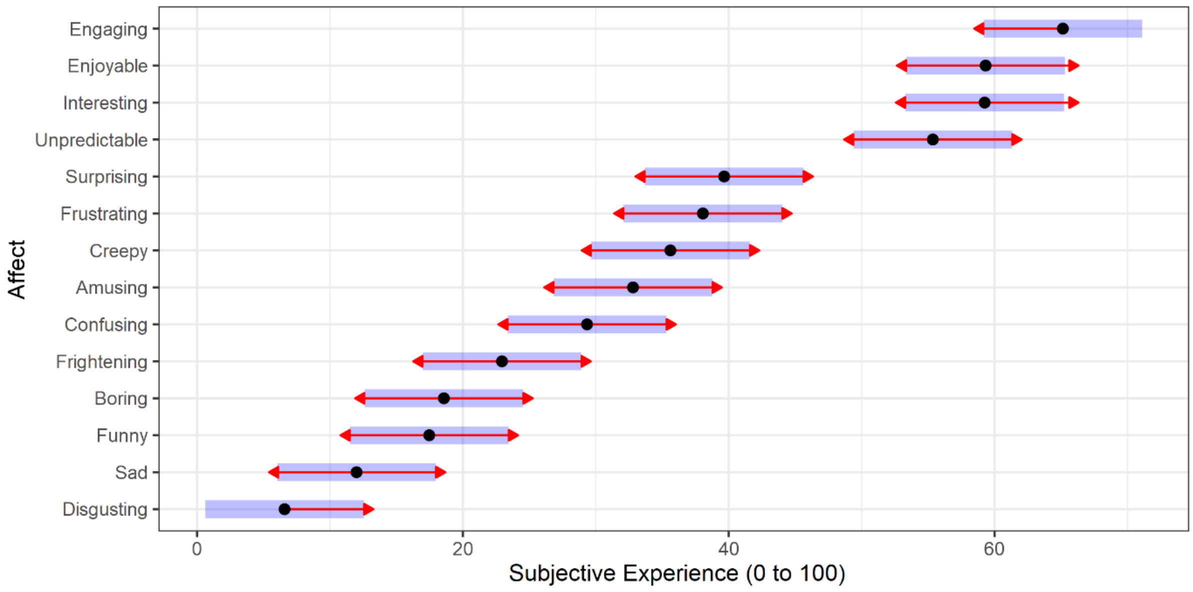

Supplementary Figure 3. Measures of affect (14 categories, scaled from 0 to 100) was used from all 70 participants that completed the VRIGT. Means (black dots), CIs (purple line) for ratings of the overall experience of the VRIGT. The red arrows highlight the comparisons between means. Overlapping arrows between two means indicate a difference is not significant based on the adjusted p-values.

User Experience Scale

The User Engagement Scale (UES) was developed to measure self-reported user engagement [1]. A sample item from this scale was "I lost myself in this experience". Responses were recorded on a 5-point Likert scale (from 1 = not at all characteristic of my experience to 5 = entirely characteristic of my experience). Higher scores indicate greater focused attention during the VRIGT.

The User Engagement Scale (UES) was broken down into four subsections to isolate the components of: focused attention (FA), perceived usability (PU), aesthetic appeal (AA), and reward factor (RF). Study 1: UES (M =3.53, SD = .51), FA (M =3.43, SD = .80), PU (M =3.77, SD = .91), AA (M =3.10, SD = .95), and RF (M =3.78, SD = .68).

Each of these averaged reported scores was above the threshold of 3 (scaling midpoint," neither agree or disagree"), suggesting the VRIGT was generally considered engaging. Regarding focused attention specifically, this suggests some degree of immersion was experienced by participants.

Tension

We also asked participants to select a graphic that best illustrated the degree and pattern of tension (a qualitative category selection) experienced during the task. Four options where visually presented to participants, depicting tension experienced during the VRIGT.

Here, results demonstrate, n= 4 [5.71%] participants reported the tension as flat (with no change over time), n= 8 [11.42%] reported tension increasing over time, n= 6 [8.57%] decreased over time, and n= 52 [74.28%] that tension dynamically changed over time. A chi-square goodness of fit statistical test demonstrated this final category significantly accounted for most individuals' reported account of tension $\chi 2$ (3) = 91.14, p >.001, w = 1.14.

Performance

LMM Results: Performance over time.

Predictors	Estimates	CI	p
(Intercept)	−3.03	−4.64 – −1.43	<.001
Block 2	1.72	−0.22 – 3.67	.082
Block 3	4.59	2.64 – 6.53	<.001
Block 4	4.03	2.09 – 5.98	<.001
Random Effects			
σ^2	28.25		
τ_{00} ppt	10.42		
ICC	0.27		
N ppt	58		
Observations	232		
Marginal R^2 / Conditional R^2	.081 / .328		

Supplementary Table 2.

Study 2

Videos

Demonstration of the VRIGT used in Study 2 available on OSF.

Power analysis

The data we collected in Study 1 was used to estimate the sample size required via simulations [2], using the SIMR package in R [3] . The results of 1000 simulated samples suggested that using a linear mixed effect model with VRIGT score being predicted by blocks, with a random factor on participant (intercepts only) a n = 50 was required to achieve a power of .80. As this study requires the inclusion of second condition, in line with the suggestion of [4] this number was doubled.

Pre-registration

Link: https://aspredicted.org/PT3_L9V

Pre-scene instructional scripts

Threat script

"You are in a building that is slowly collapsing.

Your goal is to get as far away from the danger zone as possible.

In each room, there are four doors to choose from. Selecting a door might lead you to a room that is further from the danger zone and help you escape. However, some doors will lead you to rooms where the floor is weaker, leading the danger zone to spread in your direction and cancelling any gains in distance made.

You are being tracked so you can always view your distance from the danger zone. This means you can attempt to choose the door that best maximises your distance from the danger zone.

I won't tell you how long the task will take. You must keep choosing doors until the computer stops. The computer does not make you lose distance at random. All I can say is that you may find yourself losing distance on all of the doors, but some doors will make you lose more distance than others. You can escape if you stay away from the worst doors.

Now get ready to start your escape. Good luck. "

Nonthreat script

“You are in a building attempting to find the exit so you can meet friends in the outside car park.

Your goal is to make your way as far away from your current position as possible and find the exit.

In each room, there are four doors to choose from. Selecting a door might lead you to a room that is further from your starting position and closer to the exit. However, some doors will lead you away from your starting point, but also further from the exit, cancelling any gains in distance made.

You are being tracked so you can always view your distance from your starting point. This means you can attempt to choose the door that best maximises your distance from your start location.

I won't tell you how long the task will take, but they is no need to rush. You must keep choosing doors until the computer stops. The computer does not make you lose distance at random. All I can say is that you may find yourself losing distance on all of the doors, but some doors will make you lose more distance than others. You will find the exit if you stay away from the worst doors.

Now get ready to find the exit. Good luck. “

Subjective Experience

Affect: between condition

LMM Results: Affect between condition.

Predictors	*Estimates*	*CI*	*p*
(Intercept)	8.64	1.88 – 15.40	**.012**
affect [Creepy]	8.46	−0.54 – 17.46	.065
affect [Frustrating]	33.22	24.22 – 42.22	**<.001**
affect [Unpredictable]	42.26	33.26 – 51.26	**<.001**
affect [Engaging]	41.72	32.72 – 50.72	**<.001**
affect [Confusing]	29.46	20.46 – 38.46	**<.001**
affect [Enjoyable]	44.52	35.52 – 53.52	**<.001**
affect [Boring]	21.16	12.16 – 30.16	**<.001**
affect [Funny]	9.24	0.24 – 18.24	**.044**
affect [Amusing]	21.24	12.24 – 30.24	**<.001**
affect [Sad]	−5.60	−14.60 – 3.40	.223
affect [Disgusting]	−4.38	−13.38 – 4.62	.340
affect [Interesting]	40.50	31.50 – 49.50	**<.001**
affect [Surprising]	18.70	9.70 – 27.70	**<.001**
Condition	36.34	26.78 – 45.90	**<.001**
affect [Creepy] * Condition	−4.64	−17.37 – 8.09	.475
affect [Frustrating] * Condition	−28.34	−41.07 – −15.61	**<.001**
affect [Unpredictable] * Condition	−23.62	−36.35 – −10.89	**<.001**
affect [Engaging] * Condition	−22.92	−35.65 – −10.19	**<.001**
affect [Confusing] * Condition	−30.72	−43.45 – −17.99	**<.001**
affect [Enjoyable] * Condition	−33.78	−46.51 – −21.05	**<.001**
affect [Boring] * Condition	−45.36	−58.09 – −32.63	**<.001**

affect [Funny] * Condition	−42.26	−54.99 – −29.53	**<.001**
affect [Amusing] * Condition	−34.02	−46.75 – −21.29	**<.001**
affect [Sad] * Condition	−26.32	−39.05 – −13.59	**<.001**
affect [Disgusting] * Condition	−33.86	−46.59 – −21.13	**<.001**
affect [Interesting] * Condition	−25.22	−37.95 – −12.49	**<.001**
affect [Surprising] * Condition	−19.68	−32.41 – −6.95	**.002**
**Random Effects**			
σ^2	526.59		
$\tau_{00\ PPT}$	66.64		
ICC	0.11		
N_{PPT}	100		
Observations	1400		
Marginal R^2 / Conditional R^2	.373 / .443		

Supplementary Table 3.

Pairwise comparisons: Affect between conditions.

Affect	Estimate	DF	t.ratio	P
**Frightening**	**-36.34**	**1178.667**	**-7.46007**	>.001
**Creepy**	**-31.7**	**1178.667**	**-6.50755**	>.001
Frustrating	-8	1178.667	-1.64228	.758
Unpredictable	-12.72	1178.667	-2.61123	.118
Engaging	-13.42	1178.667	-2.75493	.079
Confusing	-5.62	1178.667	-1.1537	.979
Enjoyable	-2.56	1178.667	-0.52553	.999
Boring	9.02	1178.667	1.851675	.590
Funny	5.92	1178.667	1.21529	.967
Amusing	-2.32	1178.667	-0.47626	.999
Sad	-10.02	1178.667	-2.05696	.422
Disgusting	-2.48	1178.667	-0.50911	.999
Interesting	-11.12	1178.667	-2.28277	.267
**Surprising**	**-16.66**	**1178.667**	**-3.42006**	**.009**

Supplementary Table 4. The MVT correction was applied to adjust for multivariate comparisons.

Affect: within condition

Measures of affect (14 categories, scaled from 0 to 100) were used from all participants that completed the VRIGT in the threat condition. Means (black dots), CIs (purple line) for ratings of the overall experience of the VRIGT. A LMM was used to analyse the relationship between the measures of affect used by participants to report their overall feeling during the VRIGT. The model predicted rating with a fixed effect of affect category (e.g., “frightening”). Intercepts were allowed to vary as a random factor at the level of the individual. The affect category with the lowest average rating score (“disgusting”) was used as a reference level. A significant effect of affect category was found ($F(13, 637) = 35.93$, $p < .001$).

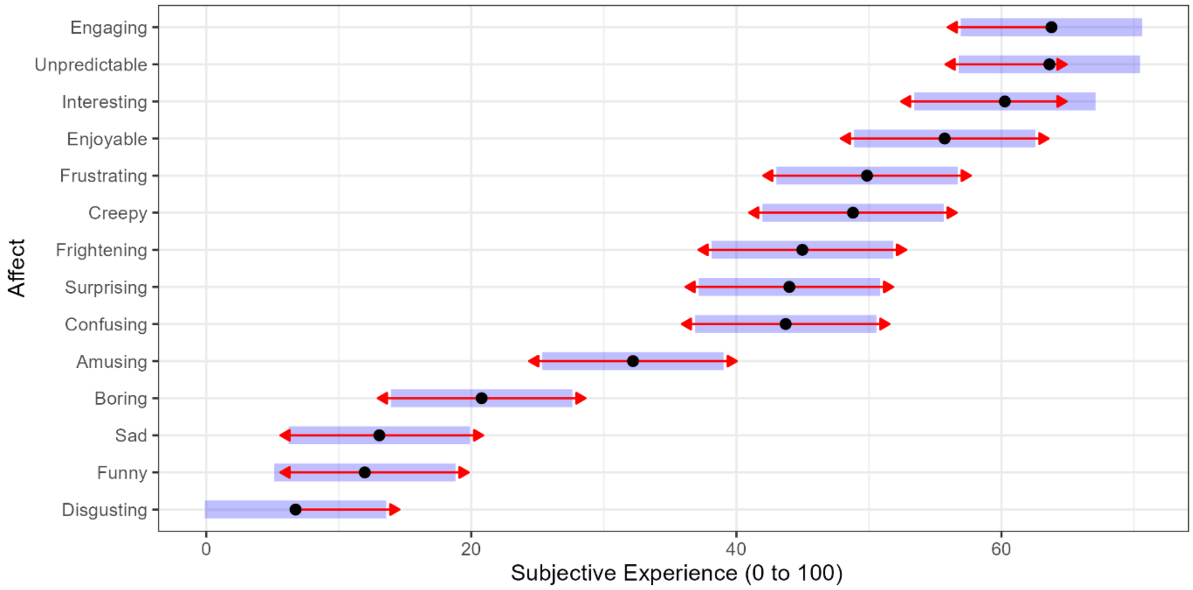

Supplementary Figure 4. Pairwise comparisons are reported using the Tukey correction for p-values highlighted significant contrasts between affect categories. The red arrows highlight the comparisons between means. Overlapping arrows between two means indicate a difference is not significant based on the adjusted p-values.

Measures of affect (14 categories, scaled from 0 to 100) were used from all participants that completed the VRIGT in the nonthreat condition. Means (black dots), CIs (purple line) for ratings of the overall experience of the VRIGT. A LMM was used to analyse the relationship between the measures of affect used by participants to report their overall feeling during the VRIGT. The model predicted rating with a fixed effect of affect category (e.g., “frightening”). Intercepts were allowed to vary as a random factor at the level of the individual. The affect category with the lowest average rating score (“sad”) was used as a reference level. A significant effect of affect category was found ($F(13, 637) = 31.02$, $p < .001$).

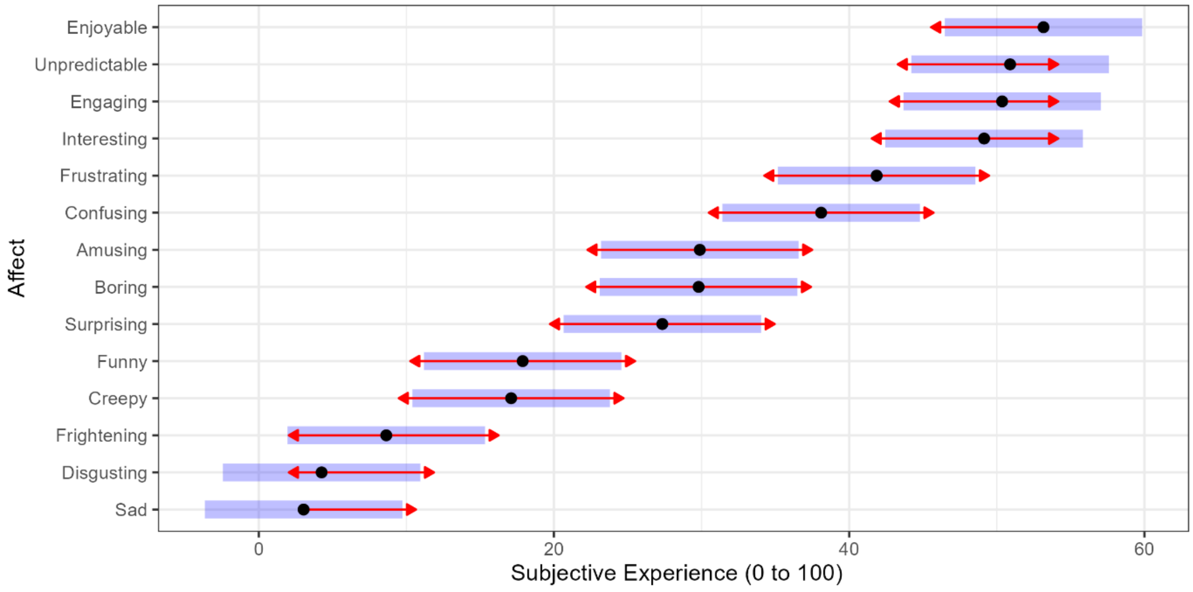

Supplementary Figure 5. Pairwise comparisons are reported using the Tukey correction for p-values highlighted significant contrasts between affect categories. The red arrows highlight the comparisons between means. Overlapping arrows between two means indicate a difference is not significant based on the adjusted p-values.

User Experience Scale

In Study 2, we only collected data on focused attention using the UES: FA, threat ($M = 3.71$, $SD = .75$), nonthreat ($M=3.62$, $SD = .71$). Each of these averaged reported scores were above the threshold of 3 (scaling midpoint," neither agree or disagree"), suggesting some degree of immersion was experienced by participants. However, we found no significant difference between conditions, $t(98) = -.594$, $p = .554$.

Tension

In the threat condition, n= 2 [4%] participants reported the tension as flat (with no change over time), n= 1 [2%] reported tension increasing over time, n= 14 [28%] decreased over time, and n= 33 [66%] that tension dynamically changed over time. While, In the nonthreat condition, n= 11 [22%] participants reported the tension as flat (with no change over time), n= 5 [10%] reported tension increasing over time, n= 16 [32%] decreased over time, and n= 18 [36%] that tension dynamically changed over time.

Performance

LMM Results: Performance over time by condition

Predictors	Estimates	CI	p
(Intercept)	−5.92	−8.23 – −3.61	<.001
Blocks 2	5.32	2.65 – 7.99	<.001
Blocks 3	6.24	3.57 – 8.91	<.001
Blocks 4	8.24	5.57 – 10.91	<.001
Blocks 5	8.88	6.21 – 11.55	<.001
Condition	0.80	−2.46 – 4.06	.630
Blocks 2 * Condition	−2.76	−6.54 – 1.02	.152
Blocks 3 * Condition	−5.52	−9.30 – −1.74	.004
Blocks 4 * Condition	−3.08	−6.86 – 0.70	.110
Blocks 5 * Condition	−4.76	−8.54 – −0.98	.014
Random Effects			
σ2	46.29		
τ00 PPT	22.53		
ICC	.33		
N PPT	100		
Observations	500		
Marginal R2 / Conditional R2	.108 / .400		

Supplementary Table 5.

Figure: Nonthreat condition, Performance score over time.

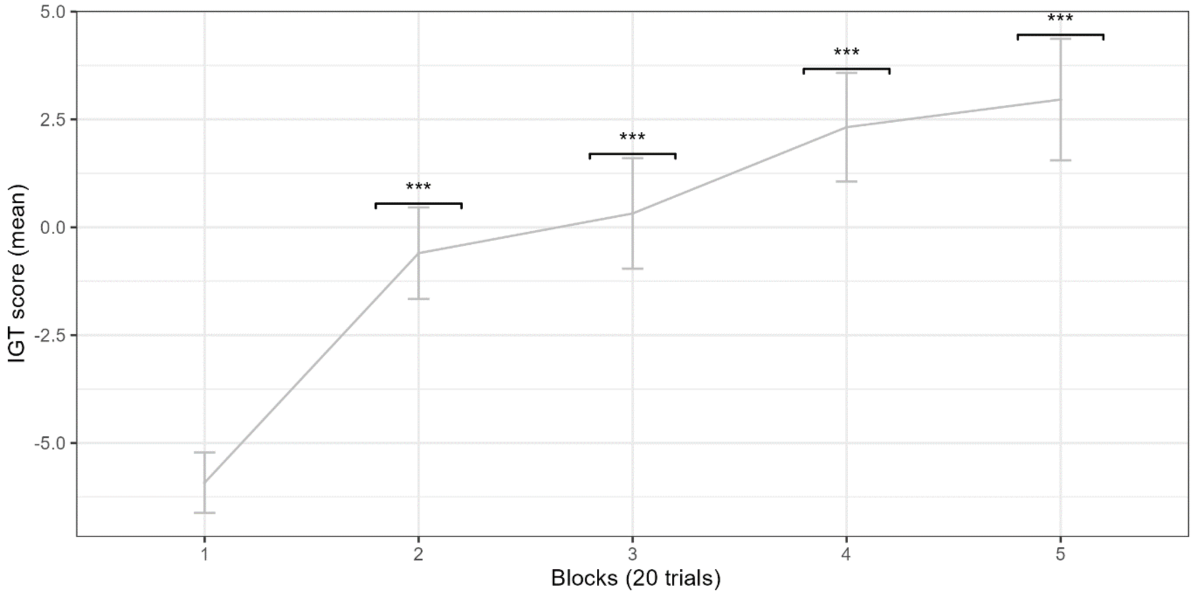

Supplementary Figure 6. Performance score over time in the nonthreat condition. Note. Error bars represent −/+ standard error. *Indicates differences from baseline from with a significant p value *< .050, ** < .01, *** <.001.

LMM Results: Performance over time in nonthreatening condition

Predictors	Estimates	CI	p
(Intercept)	−5.92	−8.22 – −3.62	<.001
Blocks 2	5.32	2.59 – 8.05	<.001
Blocks 3	6.24	3.51 – 8.97	<.001
Blocks 4	8.24	5.51 – 10.97	<.001
Blocks 5	8.88	6.15 – 11.61	<.001
Random Effects			
σ2	48.06		
τ00 PPT	20.02		
ICC	.29		
N PPT	50		
Observations	250		
Marginal R2 / Conditional R2	.127 / .384		

Supplementary Table 6.

Figure: Threat condition, Performance score over time.

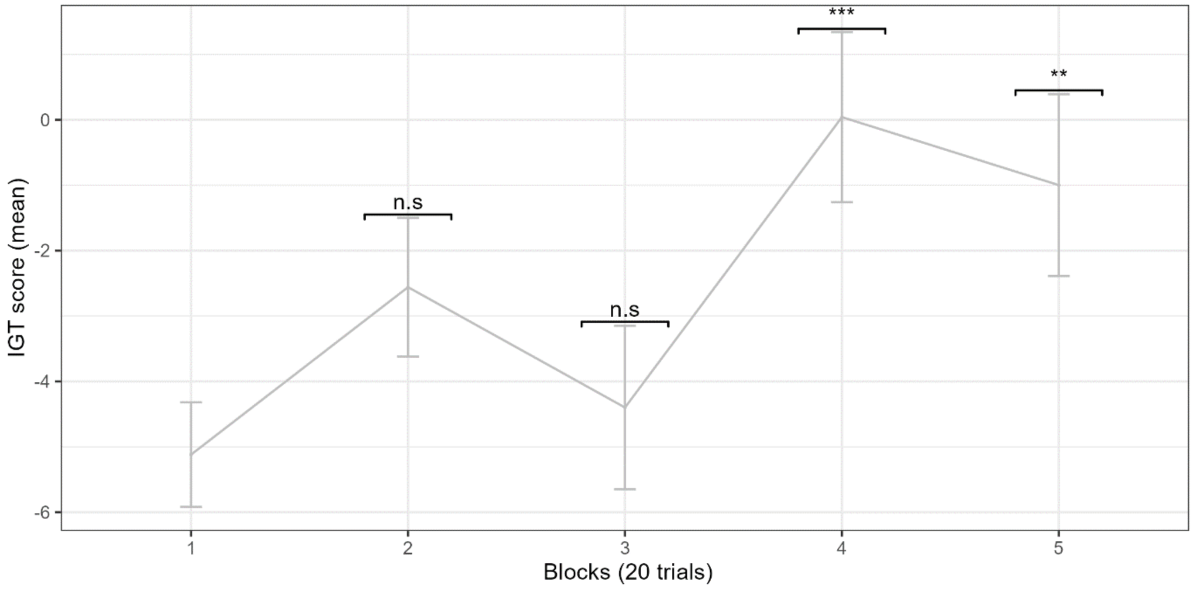

Supplementary Figure 7. Performance score over time in the threat condition. Error bars represent −/+ standard error. *Indicates differences from baseline from with a significant p value *< .050, ** < .01, *** <.001.

LMM Results: Performance over time in threatening condition

Predictors	Estimates	CI	p
(Intercept)	−5.12	−7.44 – −2.80	<.001
Block 2	2.56	−0.07 – 5.19	.056
Block 3	0.72	−1.91 – 3.35	.590
Block 4	5.16	2.53 – 7.79	<.001
Block 5	4.12	1.49 – 6.75	.002
Random Effects			
σ2	44.52		
τ00 PPT	25.03		
ICC	.36		
N PPT	50		
Observations	250		
Marginal R2 / Conditional R2	.052 / .393		

Supplementary Table 7.

Computational modelling

Figure: Nonthreat condition, posterior distributions of the hyper (group) parameters

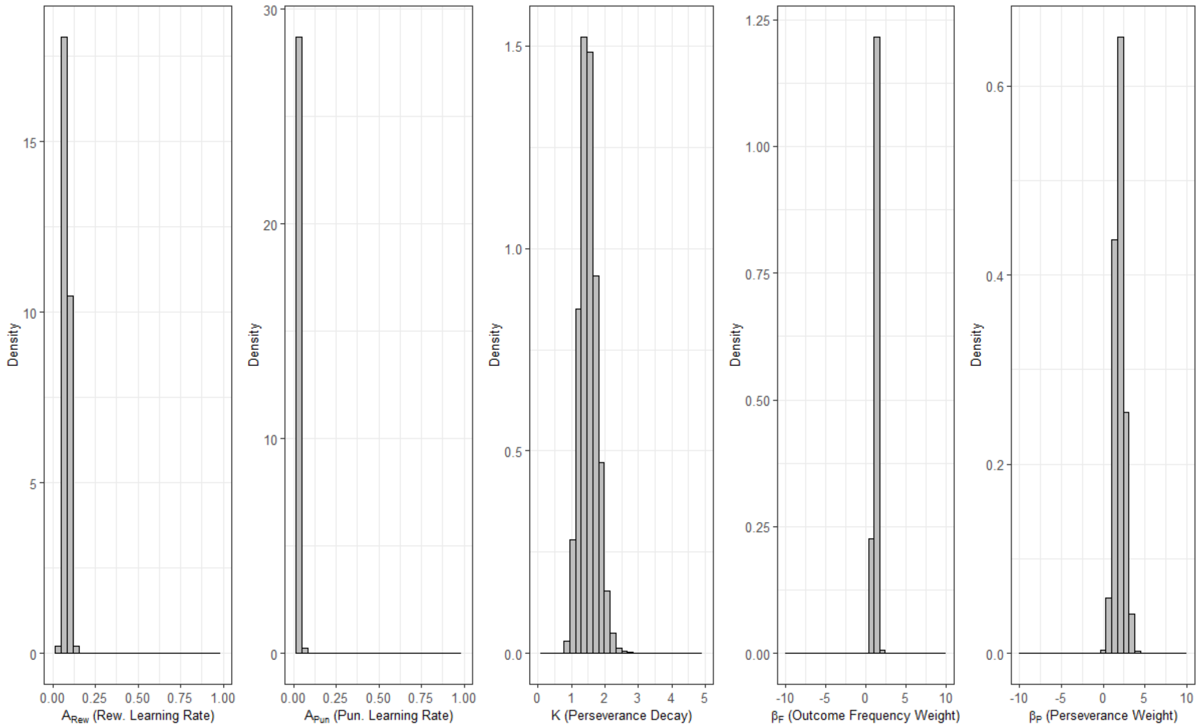

Supplementary Figure 8. Arew (Reward sensitivity), Apun (Loss sensitivity), K (Forgetfulness), betaF (Frequency sensitivity), betaP (Choice perseveration).

Figure: Nonthreat condition condition, posterior predictive checks

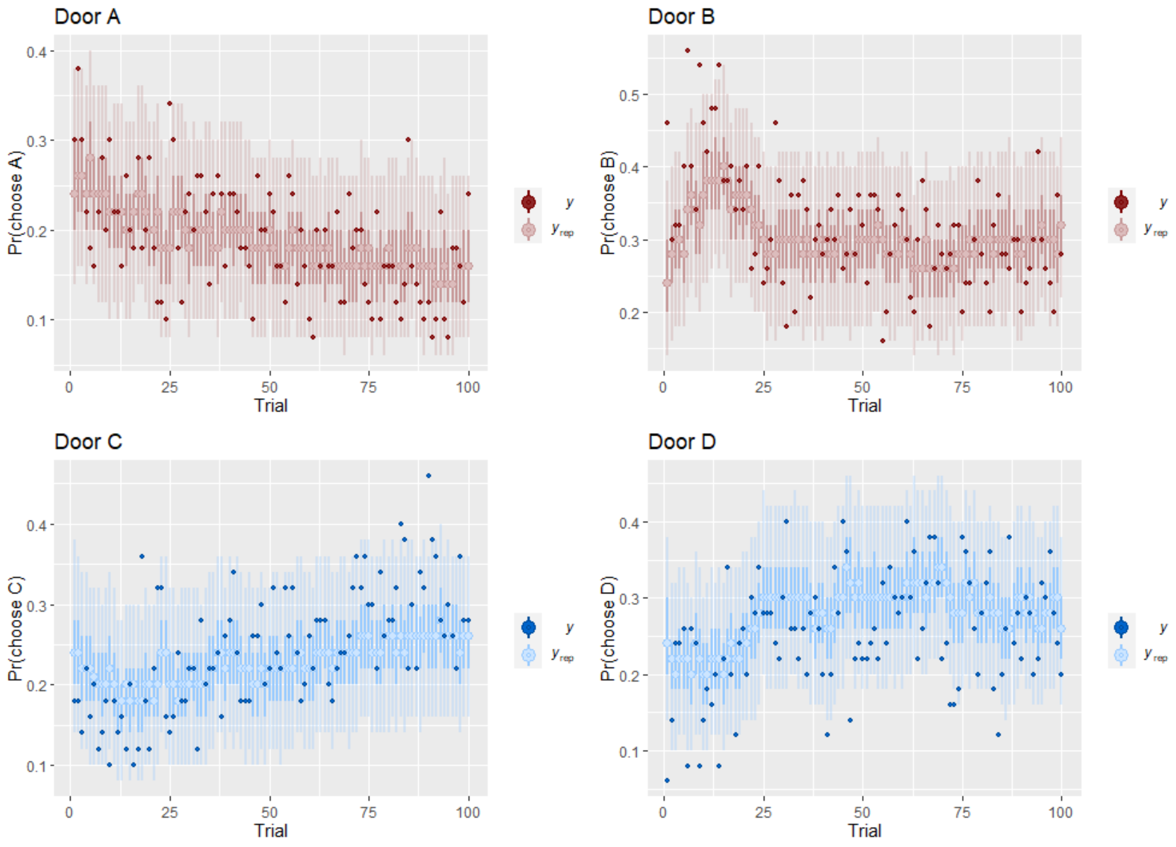

Supplementary Figure 9. Observed versus the posterior predicted probability of choosing each deck over trials. To illustrate performance trends “disadvantageous” doors are presented as red and “advantageous” doors in blue. The x-axis represents trial number, and y-axis the proportion of participants who selected the deck on each trial (Y), along with predictions (Yrep) computed from draws from the posterior predictive distribution of the same values. 50% (dark) and 95% (light) prediction intervals illustrate uncertainty.

Figure: Threat condition, posterior distributions of the hyper (group) parameters

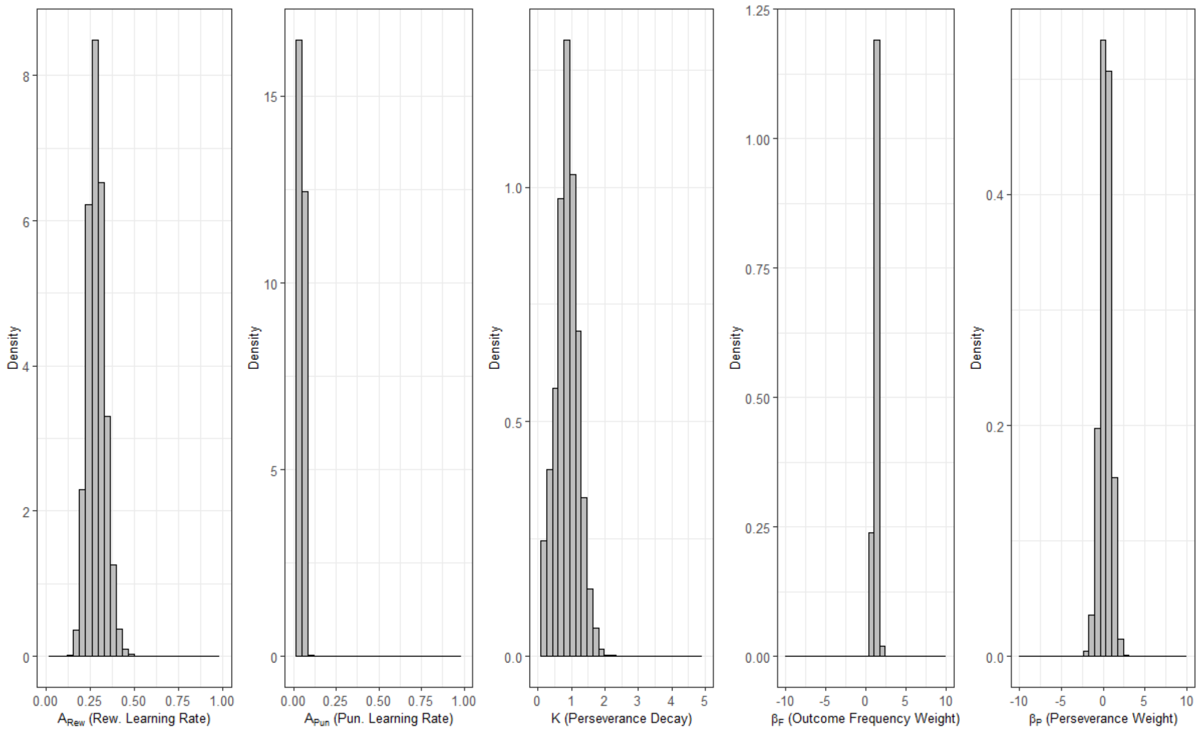

Supplementary Figure 10. Arew (Reward sensitivity), Apun (Loss sensitivity), K (Forgetfulness), betaF (Frequency sensitivity), betaP (Choice perseveration).

Figure: Threat condition, posterior predictive checks

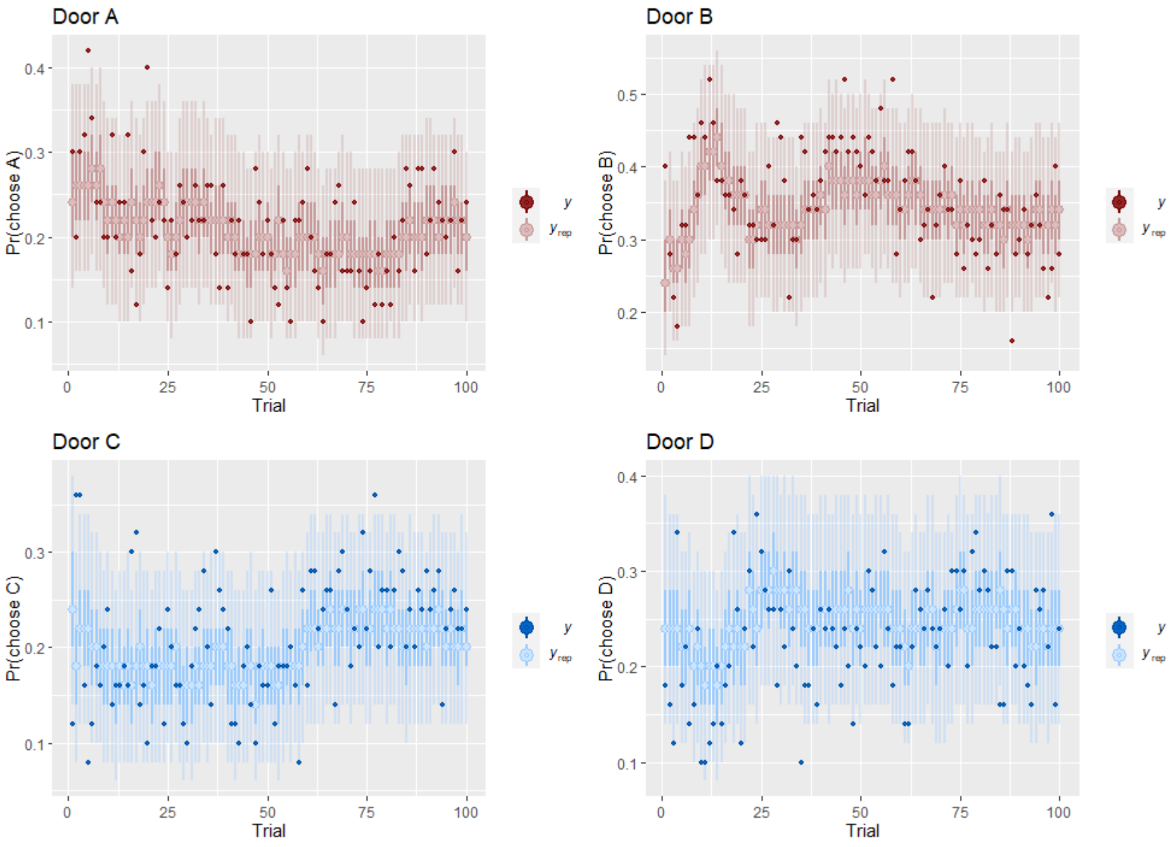

Supplementary Figure 11. Observed versus the posterior predicted probability of choosing each deck over trials. To illustrate performance trends “disadvantageous” doors are presented as red and “advantageous” doors in blue. The x-axis represents trial number, and y-axis the proportion of participants who selected the deck on each trial (Y), along with predictions (Yrep) computed from draws from the posterior predictive distribution of the same values. 50% (dark) and 95% (light) prediction intervals illustrate uncertainty.

Miscellaneous

OSF link

Follow: https://osf.io/jg2qv/?view_only=0d42f9fce5d0466685e205fde92354d2

References

1. O'Brien, H. L., Cairns, P. & Hall, M. A practical approach to measuring user engagement with the refined user engagement scale (UES) and new UES short form. *International Journal of Human-Computer Studies* **112**, 28–39 (2018).
2. Kumle, L., Võ, M. L.-H. & Draschkow, D. Estimating power in (generalized) linear mixed models: An open introduction and tutorial in R. *Behav Res* **53**, 2528–2543 (2021).
3. Green, P. & MacLeod, C. J. simr: an R package for power analysis of generalised linear mixed models by simulation. *Methods in Ecology and Evolution* **7**, 493–498 (2016).
4. Brysbaert, M. & Stevens, M. Power Analysis and Effect Size in Mixed Effects Models: A Tutorial. *Journal of Cognition* **1**, 9 (2018).
